# Addiction of lung cancer cells to GOF p53 is promoted by up-regulation of epidermal growth factor receptor through multiple contacts with p53 transactivation domain and promoter

**DOI:** 10.18632/oncotarget.6998

**Published:** 2016-01-25

**Authors:** Catherine A. Vaughan, Isabella Pearsall, Shilpa Singh, Brad Windle, Swati P. Deb, Steven R. Grossman, W. Andrew Yeudall, Sumitra Deb

**Affiliations:** ^1^ Department of Biochemistry and Molecular Biology, Virginia Commonwealth University, Richmond, VA, USA; ^2^ Massey Cancer Center, Virginia Commonwealth University, Richmond, VA, USA; ^3^ Integrated Life Sciences Program, Virginia Commonwealth University, Richmond, VA, USA; ^4^ Philips Institute, Virginia Commonwealth University, Richmond, VA, USA; ^5^ Division of Hematology, Oncology and Palliative Care, Virginia Commonwealth University, Richmond, VA, USA; ^6^ Department of Oral Biology, Augusta University, Augusta, GA, USA

**Keywords:** addiction, mutant, p53, transcription, EGFR

## Abstract

Human lung cancers harboring gain-of-function (GOF) p53 alleles express higher levels of the epidermal growth factor receptor (EGFR). We demonstrate that a number of GOF p53 alleles directly upregulate EGFR. Knock-down of p53 in lung cancer cells lowers EGFR expression and reduces tumorigenicity and other GOF p53 properties. However, addiction of lung cancer cells to GOF p53 can be compensated by overexpressing EGFR, suggesting that EGFR plays a critical role in addiction. Chromatin immunoprecipitation (ChIP) using lung cancer cells expressing GOF p53 alleles showed that GOF p53 localized to the EGFR promoter. The sequence where GOF p53 is found to interact by ChIP seq can act as a GOF p53 response element. The presence of GOF p53 on the EGFR promoter increased histone H3 acetylation, indicating a mechanism whereby GOF p53 enhances chromatin opening for improved access to transcription factors (TFs). ChIP and ChIP-re-ChIP with p53, Sp1 and CBP histone acetylase (HAT) antibodies revealed docking of GOF p53 on Sp1, leading to increased binding of Sp1 and CBP to the EGFR promoter. Up-regulation of EGFR can occur via GOF p53 contact at other novel sites in the EGFR promoter even when TAD-I is inactivated; these sites are used by both intact and TAD-I mutated GOF p53 and might reflect redundancy in GOF p53 mechanisms for EGFR transactivation. Thus, the oncogenic action of GOF p53 in lung cancer is highly dependent on transactivation of the EGFR promoter via a novel transcriptional mechanism involving coordinated interactions of TFs, HATs and GOF p53.

## INTRODUCTION

Wild-type (WT) p53 acts as a tumor suppressor protein, yet single amino acid substitutions prevalent in many cancers, including lung cancer, abrogate the tumor suppressor function and endow it with dominant proliferative and oncogenic properties (gain of function, GOF). p53 is found to be mutated at a high frequency (for example, 30% in non-small cell lung carcinoma to 70% in small cell lung carcinoma) [1, 2]. GOF p53, in general, is expressed at a relatively high level in cancer cells, while WT p53 is found only in low amounts in unstressed normal cells. Clinical and laboratory studies suggest that lung cancers with p53 mutations carry a worse prognosis and are more resistant to chemotherapeutic drugs and radiation [3, 4].

In laboratory settings, a number of different phenotypes have been ascribed to the GOF activities of GOF p53 including increased tumorigenicity [5, 6], decreased sensitivity to chemotherapeutic drugs [4, 7, 8], increased growth rate [9], and increased motility [10], amongst others. The molecular mechanism behind GOF revolves around two mutually non-exclusive concepts. One involves transcriptional modulation of target genes by tumor-derived GOF p53. For example, GOF p53 may transactivate growth-promoting or anti-apoptotic genes, or even growth suppressive genes [11, 12]. Our gene expression analyses provide evidence for this [7, 9]. The other concept implicates protein-protein interactions between GOF p53 and other cellular protein(s) such as the p53 family members, p63 and p73 [13], or interference with AMPK [14].

We and others have demonstrated that GOF p53 upregulates a series of genes, especially those involved in cell proliferation and oncogenesis [9, 15, 16]. In earlier work, we showed that GOF p53 transactivates the human epidermal growth factor receptor (EGFR) promoter in transient transfection assays in the absence of specific DNA binding by p53 [17, 18]. EGFR is involved in cell proliferation and motility [19] and its over-expression has been found to be implicated in various cancers including lung cancer [20]. The mechanism through which GOF p53 upregulates gene expression is, however, not yet clear.

In this communication, we show that lung cancer cells expressing GOF p53 are addicted to GOF p53; knock-down of p53 causes lowering of tumorigenicity and other GOF properties. We demonstrate that GOF p53 upregulates EGFR expression and activates the EGFR pathway. Knock-down of p53 lowers EGFR over-expression; however, the addiction to GOF p53 can be compensated by overexpressing EGFR, suggesting that EGFR is in the GOF p53 pathway and plays a critical role in the addiction of lung cancer cells to GOF p53. Using chromatin immunoprecipitation (ChIP) assays we show that GOF p53 interacts with the EGFR promoter and increases H3 histone acetylation. ChIP and ChIP-re-ChIP studies show docking of GOF p53 on Sp1 as well as increased binding of Sp1 and CBP on the EGFR promoter. We propose a model in which GOF p53 binds to the target promoter, recruits a TF and increases histone acetylation by associating with a factor like CBP, thus inducing chromatin opening for further promoter-TF interactions.

## RESULTS

### Lung tumor cells expressing GOF p53 show higher EGFR levels

**Figure 1:**
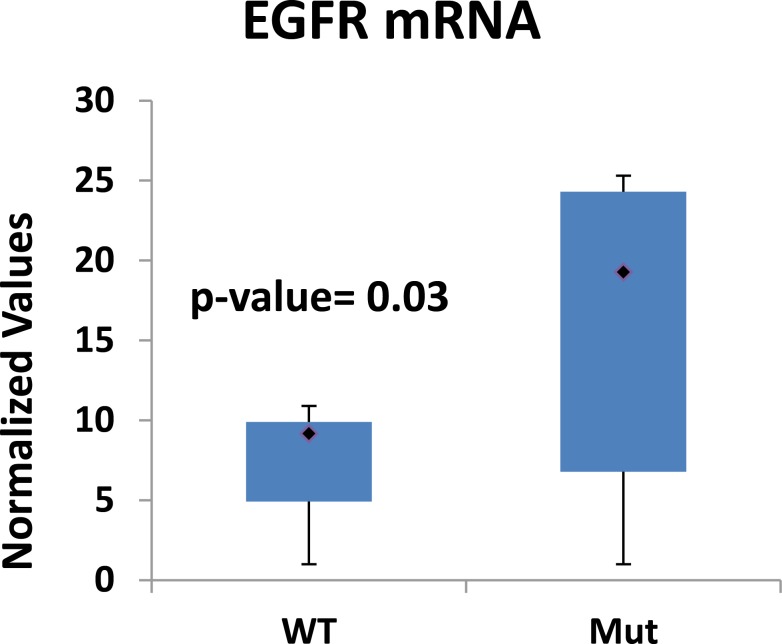
Lung tumor cells expressing GOF p53 show higher EGFR levels RT-QPCR of EGFR levels in lung tumors. cDNA was prepared from human lung tumor RNAs using the Superscript III cDNA synthesis kit (Invitrogen) and QPCR performed using primers specific for EGFR. The degree of expression was quantitated using a relative standard curve, normalized to GAPDH corresponding to the cDNA batch, and presented as a box plot to show the distribution of EGFR expression in WT and GOF p53 containing lung tumors. We used 15 NSCLC lung tumor samples for each set of either WT or mutant p53. Mutations within the samples were found mostly within the DNA binding domain (DBD) with a few located within the oligomerization domain. Experiments were performed in technical triplicates as described in the text. Error bars showing standard deviations are indicated and the p-value has been included.

Since we previously observed that GOF p53 transactivates the EGFR promoter [18], we tested if expression of EGFR is upregulated in human lung tumors expressing GOF p53. Figure 1 depicts EGFR mRNA levels of different human lung tumors collected in Virginia Commonwealth University's cancer tissue repository. On average, there was more EGFR expression in samples with GOF p53 *versus* samples with WT p53 (average 2.1-fold, p-value 0.03), corroborating our cell culture data that GOF p53 up-regulates EGFR expression. Thus, overall there is an increased expression of EGFR in human lung tumors with GOF p53.

**Figure 2:**
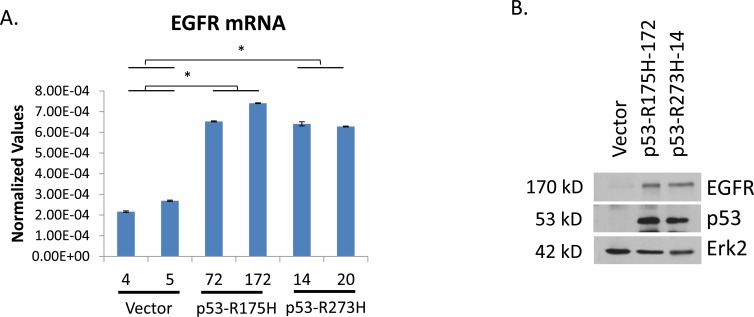
Gain-of-function p53 upregulates expression of EGFR in H1299 lung cancer cells H1299 cells have been stably transfected to express p53 mutants -R175H and -R273H (or vector alone). **A.** RT-QPCR was used to assay for EGFR levels in different cell clones. The data presented show that GOF p53 upregulates EGFR mRNA expression. Data represent QPCR values normalized to GAPDH levels (that are not affected by GOF p53). Different cell clones are indicated by clone numbers. Experiments were performed in technical triplicates. Error bars showing standard deviations are indicated. Asterisks indicate a p-value of less than 0.05. **B.** Representative Western analysis showing EGFR and GOF p53 levels in different cell clones.

Tumor-derived GOF p53 induces expression of the EGFR gene. Once we found that GOF p53 binds to the EGFR promoter region, coupled with our knowledge that GOF p53 also transactivates the EGFR promoter [17, 18], we tested whether H1299 cells expressing p53-R175H and -R273H show higher levels of EGFR mRNA compared to vector transfected cells. We prepared RNA from these cells and determined EGFR mRNA levels in samples prepared from two individual clones per transfection. Figure 2 demonstrates that EGFR expression is up-regulated by the p53 mutants in each case in multiple stable clones. Figure 2B shows an example of a Western blot with higher level of EGFR in H1299 cells expressing p53 mutants.

**Figure 3:**
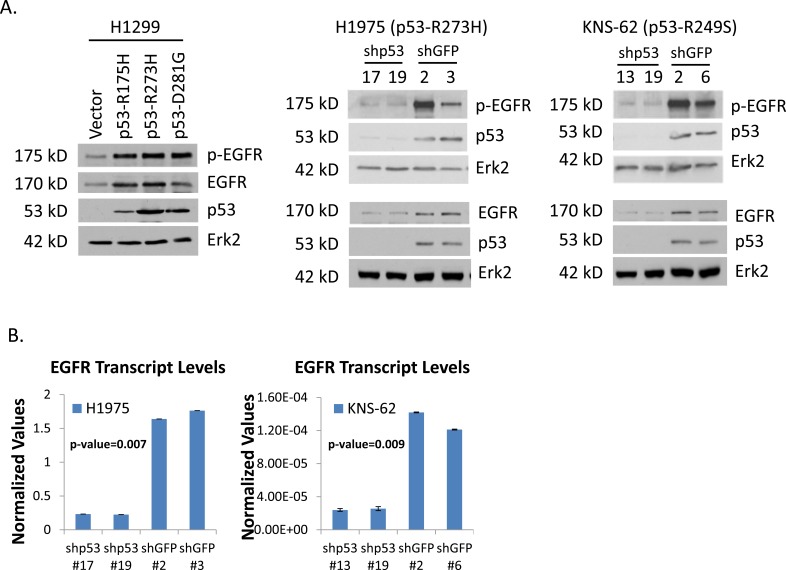
p53 knock-down in H1975 and KNS-62 cells reduces EGFR levels **A.** Western blot showing EGFR, phospho-EGFR, p53, and Erk2 levels in H1299 cells stably expressing either an empty vector or the p53 mutants R175H, R273H, and D281G as well as different cell clones generated by recombinant lentiviruses expressing p53 shRNA or control GFP shRNA in H1975 and KNS-62 cells. **B.** RT-QPCR data for EGFR in different cell lines under study. cDNA was prepared from cell line RNAs using the Superscript III cDNA synthesis kit (Invitrogen) and QPCR performed using primers specific for EGFR cDNA. The degree of expression was quantitated using a relative standard curve and normalized to GAPDH corresponding to the cDNA batch. Different cell clones are indicated by clone numbers. Experiments were performed in technical triplicates. Error bars showing standard deviations are indicated and p-values have been included.

EGFR is a target of GOF p53. Next, we wanted to determine if EGFR behaves as a GOF p53 inducible gene in lung cancer cells expressing endogenous GOF p53. Thus, we generated p53 knocked-down derivatives from lung cancer cells H1975 (p53-R273H) and KNS-62 (p53-R249S) using lentiviral vectors carrying p53 shRNA. Figure 3 indicates knock-down of the endogenous p53 in stable clones of H1975 and KNS-62 cell lines and shows that the EGFR level is reduced upon GOF p53 knock-down consistent with EGFR being a GOF p53 target gene. Figure 3B shows the results of RT-QPCR experiments to assay for EGFR levels in the cell clones generated (Figure 3A).

Since GOF p53 transactivates the EGFR promoter and induces EGFR expression, we tested whether it results in enhanced phosphorylation of EGFR, which is indicative of the activation of EGFR pathway [19]. We tested the level of these proteins in H1299 cells expressing different p53 mutants (or vector control). Data presented in Figure 3A show that expression of p53-R175H, -R273H, and -D281G led to an increase of phospho-EGFR. These data are corroborated by our observations in H1975 and KNS-62 p53 knock-down (and GFP knock-down control) cells.

**Figure 4:**
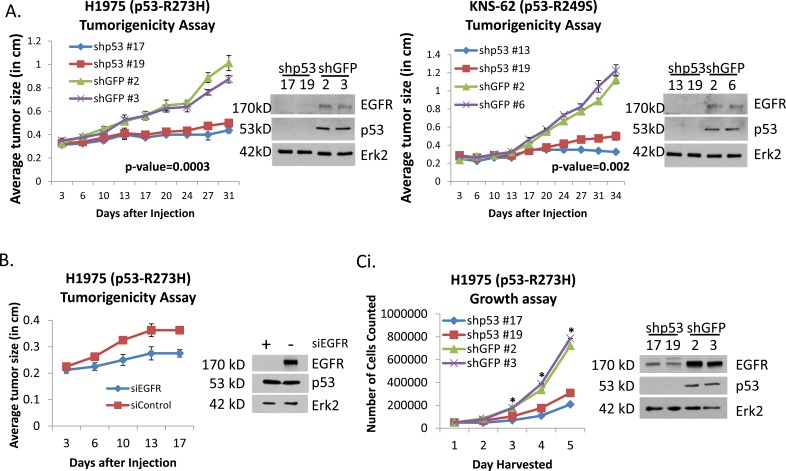

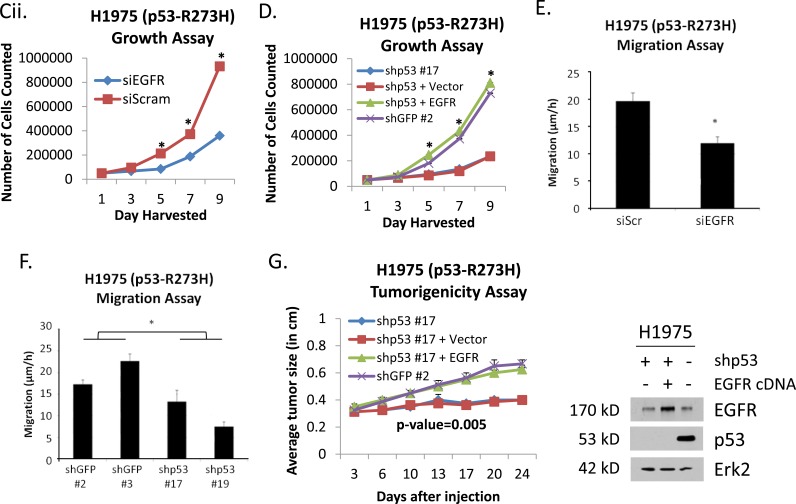
Reduction of GOF p53 and EGFR in lung cancer cells retards tumorigenicity, growth rate and cell motility A. H1975 and KNS-62 cell clones stably expressing shRNA against p53 were injected into nude (H1975) or Scid (KNS-62) mice. Western blot analysis was performed on extracts derived from tumors after removal from the mice. B. H1975 lung cancer cells were transfected with siRNA targeting EGFR and subsequently injected into nude mice. Western blot analysis was performed on extracts derived from tumors after removal from the mice and used to show knoc-kdown of the EGFR protein in 4B, 4Cii, and 4E. C. (i) Growth assay of H1975 cells knocked-down for p53 (and control) generated by recombinant lentivirus expressing p53 shRNA. Western blot analysis was performed to show knock-down of the EGFR protein in 4Ci and 4F. (ii) Growth rate of H1975 cells depends on the EGFR level. H1975 cells were transfected with control or EGFR-specific siRNA, plated in equal numbers, and harvested every 48 hours for five time points to determine the rate of doubling. Asterisks indicate a p-value of less than 0.05. D. H1975 p53 knock-down cells were transfected with an EGFR expression plasmid to compensate for the EGFR expression loss, and a growth assay was performed. The assay has been described in the text. A representative immunoblot showing the level of EGFR is shown in 4G. Asterisks indicate a p-value of less than 0.05. E. Migration of H1975 after transient transfection of RNAi against EGFR shows a reduced migration rate. Asterisk indicates a p-value of less than 0.05. F. H1975 cells show a reduction in migration when the endogenous GOF p53 is stably knocked-down. Asterisk indicates a p-value of less than 0.05. G. H1975 p53 knock-down cells were transfected with an EGFR expression plasmid to compensate for the EGFR expression loss, and tumorigenicity (in nude mice) assays were performed. Nude mice were injected with following cell systems: I. H1975 shGFP, (ii) H1975 shp53, (iii) H1975 shp53 + vector and (iv) H1975 shp53 + EGFR. EGFR expression recovers GOF activity loss observed on knock-down of GOF p53 of H1975 cells. Western blot showing EGFR and p53 levels in tumors that were resected from nude mice is shown. Experiments were performed in triplicate. Error bars showing standard deviations are indicated. The p-value has been indicated.

Lung cancer cells with endogenous GOF p53 are addicted to GOF p53. We tested whether reduction of p53 would cause significant reduction in oncogenic functions of lung cancer cells as measured by tumorigenicity in immunodeficient mice. Thus, we performed tumorigenicity assays in nude or Scid mice as described in Materials and Methods. Figure 4A shows that p53 knock-down in H1975 and KNS-62 cells results in remarkable reduction of tumorigenicity, demonstrating that these lung cancer cells are addicted to GOF p53 for effective tumor formation.

Reduction of GOF p53 and EGFR in lung cancer cells retards tumorigenicity, growth rate and cell motility. We then wanted to test whether reduction of p53 can be mimicked by EGFR knock-down in terms of reduction of oncogenicity as measured by tumorigenicity as well as proliferation and motility rate of lung cancer cells. Thus, we performed growth assays as described in Materials and Methods. Similarly, we transiently transfected H1975 cells with EGFR siRNA (or scrambled siRNA) and performed nude mice tumorigenicity and cell growth assays. Tumorigenicity data shown in Figure 4B indicate a drastic effect on the tumor growth of H1975 cells suggesting a strong dependence of the growth of the tumor cells on EGFR even when GOF p53 is present. Western blot analysis performed on tumor samples indicated that the EGFR level remained lower at the time when tumors were harvested. Also, data shown in Figure 4C show that knock-down of either p53 or EGFR reduced the growth rate significantly. This result suggests that GOF p53 regulates cell growth, at least in part, through EGFR expression. In parallel, we performed wound closure assays to determine the impact of reducing GOF p53 and EGFR on cell motility. As shown in Figure 4E and 4F respectively, EGFR and GOF p53 knock-down resulted in a decrease in cell motility.

### EGFR can compensate for GOF p53 deficiency

We hypothesized that GOF p53 may execute (some of) its oncogenic functions *via* the EGFR pathway; if that is true, then the defects encountered by knock-down of GOF p53 should be compensated by EGFR over-expression in those cells. Therefore, we tested whether EGFR over-expression could restore the growth and tumorigenicity defect encountered by knock-down of GOF p53. Figure 4D and 4G show that expression of EGFR by transfection of H1975 (p53-R273H) p53 knock-down cells with an EGFR expression plasmid compensates for the reduced growth rate and, more importantly, tumorigenicity, respectively, in nude mice. Western blot analysis performed on tumor samples indicated that the EGFR level remained higher at the time when tumors were harvested. This suggests that EGFR plays a crucial role in mediating the effects of the GOF p53 GOF pathway.

**Figure 5:**
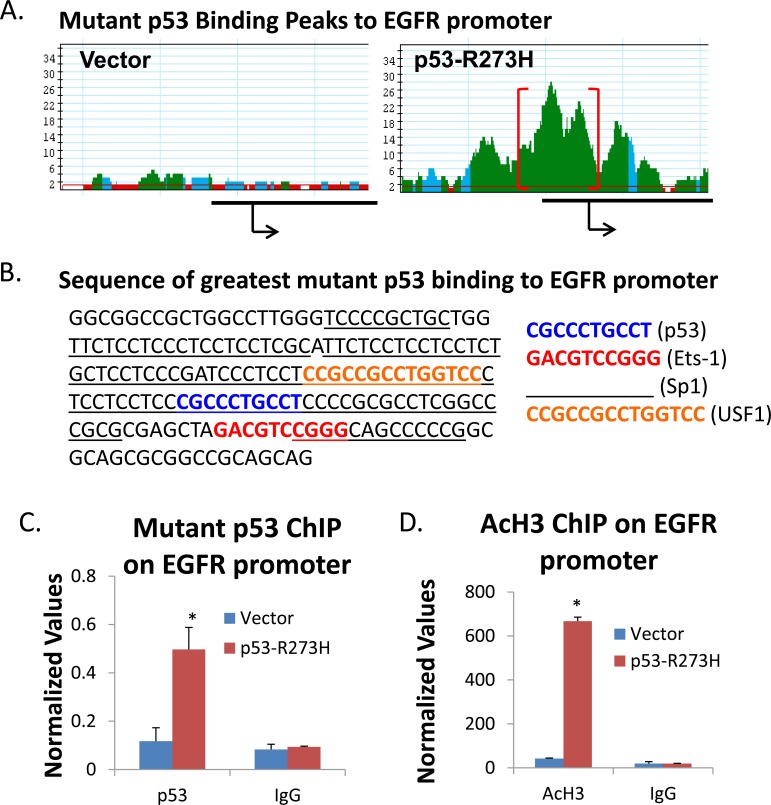

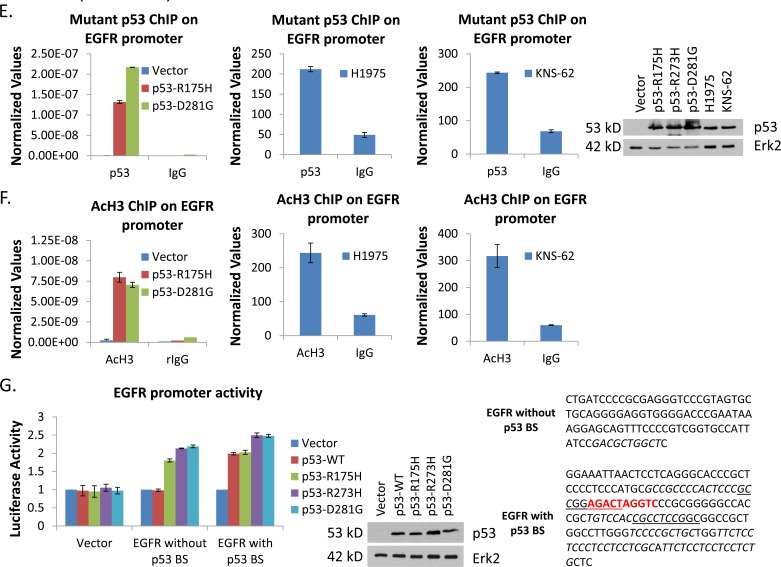

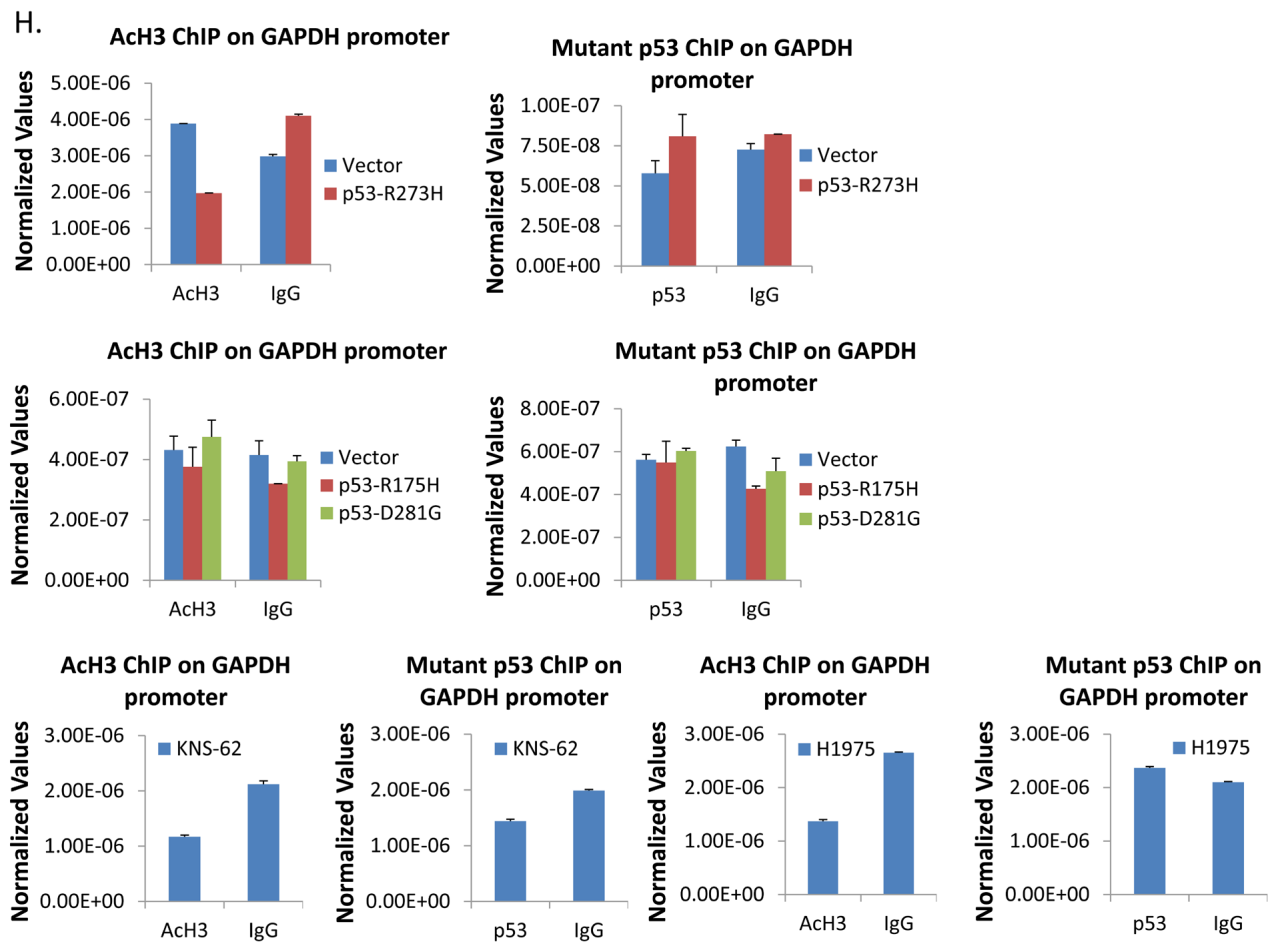

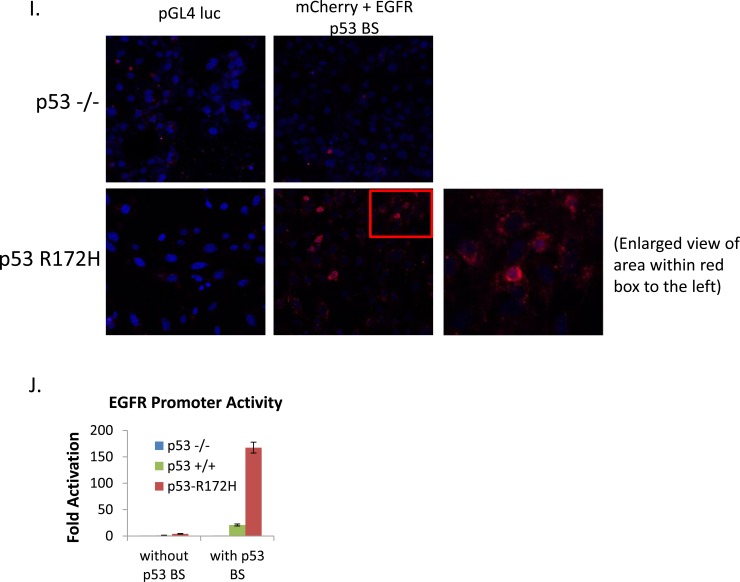
p53 ChIP sequence peaks on EGFR gene upstream sequences and QPCR verification of ChIP on the EGFR promoter Details of ChIP seq analysis and their verifications have been described previously [36]. **A.** The peaks representing areas under which maximal p53-R273H binding occurs as apparent by next generation sequence analysis. **B.** Sequence of the major peak (shown above in red brackets in A where GOF p53 binding occurs along with some of the prominent TF binding sites. **C.** and **D.** ChIP assay results showing p53-R273H and AcH3 binding to the EGFR promoter. **E.** ChIP assay result showing GOF p53 binding to the EGFR promoter in cells stably expressing the R175H and D281G p53 mutants as well as two lung cancer cell lines (H1975 and KNS-62) that endogenously express different p53 mutants (−R273H and -R249S respectively). **F.** ChIP assay result showing AcH3 binding to the EGFR promoter in cells stably expressing the R175H and D281G p53 mutants as well as two lung cancer cell lines (H1975 and KNS-62). **G.** Transient EGFR promoter assay in H1299 cells transfected with p53 expression plasmids for p53-WT, -R175H, -R273H, and -D281G. Two regions of the EGFR promoter were cloned into a minimal promoter vector (pGL4-luc): one that did not contain a p53 binding site (BS), and one that did. Sequences to the right show the EGFR promoter with and without the p53 binding site. The sequence in red indicates the p53 binding site, sequences underlined indicate Ets-1 transcription factor sites, and sequences in italics indicate Sp1 transcription factor sites. **H.** QPCR analysis of a negative control region on the GAPDH promoter shows that neither mutant p53 or AcH3 binds in H1299 cells stably expressing different p53 mutants or in H1975 or KNS-62. **I.** Confocal microscope images of primary lung cells derived from mice expressing p53 −/− and R172H/R172H after transfection with our mCherry-EGFR.luc expression plasmid (where the EGFR minimal promoter with and without the p53 binding site was cloned), and mounted on slides after fixation [24, 25]. Cells were viewed under a Zeiss LSM 700 confocal microscope using the 20X objective. Area within red box is enlarged to the right. **J.** Luciferase assay in cells derived from p53 −/−, p53 +/+, and p53-R172H/R172H transfected with the mCherry-EGFR.luc (minimal EGFR promoter containing a p53 binding site and the promoter without the binding site). Sequences cloned for the EGFR promoter response element are indicated in G; the p53 binding site is in red and bold. ChIP assays were carried out as described in Materials and Methods with H1299 cells expressing p53-R273H, -R175H, and -D281G and vector control as well as H1975 and KNS-62 using antibodies against p53 and AcH3. Data represent QPCR values normalized to a fragment on the GAPDH promoter not affected by GOF p53. Experiments were performed in technical triplicates. Error bars indicate standard deviations. Experiments were performed multiple times with similar results. Asterisks indicate a p-value of less than 0.05.

Tumor-derived GOF p53 binds on the upstream region of the EGFR gene and induces histone acetylation. In order to decipher the mechanism of activation of gene expression by GOF p53 we first identified promoter sequences bound by GOF p53-R273H in H1299 cells expressing p53-R273H by performing ChIP-Seq [to be communicated separately, [36]]. In this analysis, we identified EGFR as a candidate gene whose promoter is bound by GOF p53. Figure 5A shows GOF p53 (R273H) ChIP-Seq driven peak analysis of mutant binding on the EGFR promoter with Figure 5B giving the sequence where maximal GOF p53 binding occurs (indicated by brackets surrounding the peaks). Some of the known TF binding sites are identified in the sequence.

We verified a number of GOF p53 mutants binding on the promoter region of the EGFR gene by ChIP assays followed by QPCR (Figure 5C, 5E). We also carried out ChIP assays with an antibody against acetylated histone H3 (AcH3) (Figure 5D, 5F). Figure 5D shows increased binding of K9/K14 acetylated histone H3 to the EGFR promoter upon expression of GOF p53. We performed similar ChIP experiments with lung cancer cell lines expressing endogenous p53 mutants (H1975 and KNS-62) (Figure 5E, 5F); the data show that these p53 mutants also interact similarly (albeit less vigorously, possibly due to differences in cellular contexts between H1299 and H1975 and KNS-62 and the level of mutant p53 being somewhat lower in the lung cancer cell lines *versus* H1299 stably expressing different p53 mutants on immunoblot analysis as the ChIP assay data apparently depend on the complex formation of GOF p53 and one or more transcription factors in the cells) to those expressed in H1299 cells (Figure 5C). Similarly, AcH3 ChIP data (Figure 5F) recapitulated the data shown in Figure 5D. Figure 5H shows mutant p53 and AcH3 ChIP assays performed in H1299 cells stably expressing different p53 mutants as well as H1975 and KNS-62 lung cell lines where neither mutant p53 or AcH3 binds to a control region on the GAPDH promoter.

### Sequences from the EGFR promoter where GOF p53 interacts act as a GOF p53 response element

We have cloned the sequences shown in Figure 5B, the major GOF p53 interaction site on the EGFR promoter, upstream of the minimal SV40 promoter in the pGL4.luc construct to determine if this sequence would make the minimal promoter responsive to GOF p53. Data depicted in Figure 5G show that such is the case. This promoter gets activated by both WT and GOF p53. We also eliminated the p53 binding site identified in that region and cloned that upstream of the minimal promoter; the data presented demonstrate that the cloned sequences indeed induced GOF p53 response, but lost WT p53 response. The luciferase vector that did not have the GOF p53 interaction site did not get upregulated by WT or GOF p53 as well. Thus, the cloned sequences contain a GOF p53 response element.

To determine if the endogenous GOF p53 level is high enough to induce transactivation of the EGFR promoter, we sub-cloned the EGFR promoter responsive to mutant p53 into pGL4.luc and cloned EGFR-luc downstream of mCherry. We used this bicistronic construct as described in Materials and Methods to test transactivation by mutant p53. We transfected primary mouse lung cells isolated from p53−/− and p53-R172H/p53-R172H mice [24, 25]. Figure 5I clearly shows predominantly more fluorescence in p53-R172H/p53-R172H cells compared to p53−/− cells demonstrating transactivation by mutant p53-R172H (mouse equivalent of human p53-R175H) of the EGFR promoter. In the luciferase assays (Figure 5J) we saw that while the WT p53 present in p53 +/+ cells activated the EGFR promoter in the presence of the p53 binding site, this activation was lost when it was deleted, confirming our results in H1299 cells. Thus, our data show that GOF p53 upregulates EGFR expression.

### TFs are involved in inducing binding of acetylated histone H3 on the EGFR promoter

**Figure 6:**
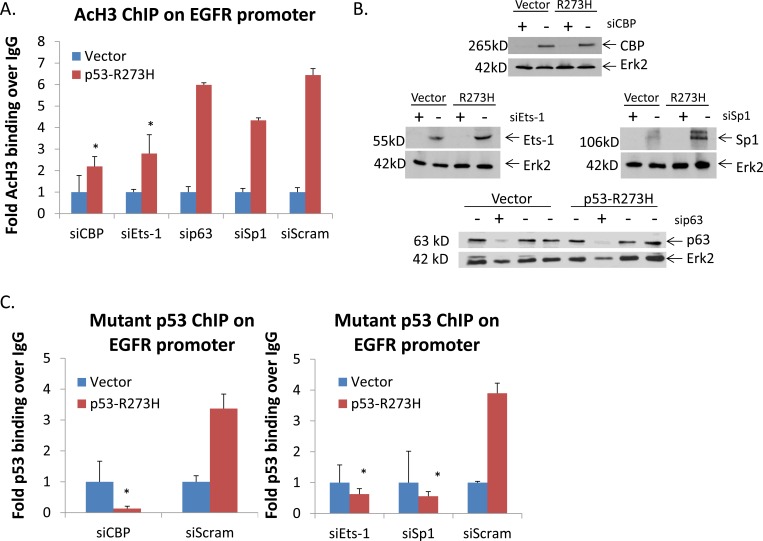

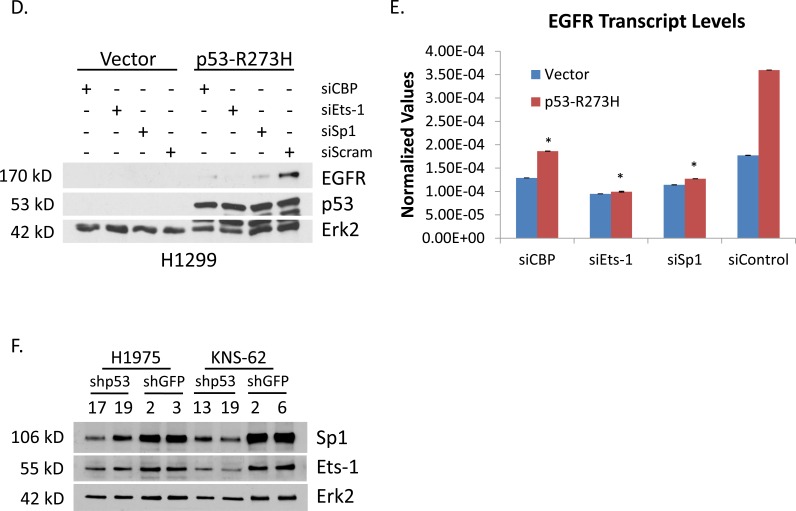
TFs are involved in inducing binding of acetylated histone H3 and p53 on the EGFR promoter H1299 cells stably expressing p53-R273H (or vector alone) were transfected with RNAi against TFs suspected of binding to the EGFR promoter (or scrambled siRNA). **A.** ChIP analysis was performed using an antibody against AcH3 as described in Materials and Methods. QPCR was performed using gene specific primers. Data represent QPCR values normalized to a region on the GAPDH promoter which is not affected by GOF p53. Normalized values for each siRNA treatment were divided by the normalized IgG value to calculate fold binding over IgG. The vector was then set to 1 in each set to be able to compare AcH3 binding between the different transcription factor knock-downs. The data show that multiple TFs influence AcH3 binding to the EGFR promoter. Experiments were performed in triplicate. Error bars showing standard deviations are indicated. Asterisks indicate a p-value of less than 0.05. **B.** Western blot shows extent of knock-down of different TF levels. **C.** ChIP assays to determine the extent of TF-mediated p53 binding on the EGFR promoter. H1299 cells expressing p53-R273H (or vector alone) were transfected with RNAi against Sp1, CBP and Ets1 (or scrambled siRNA), and ChIP analysis was performed using p53 antibodies as described in Materials and Methods. Data represent QPCR values normalized to a region on the GAPDH promoter which is not affected by GOF p53. Normalized values for each siRNA treatment were divided by the normalized IgG value to calculate fold binding over IgG. The vector was then set to 1 in each set to be able to compare p53 binding between the different transcription factor knock-downs. Error bars showing standard deviations are indicated. Asterisks indicate a p-value of less than 0.05. **D.** Western analysis of p53 and EGFR expression in siRNA treated cells used for ChIP in A and C. **E.** EGFR mRNA expression in siRNA treated cells used for ChIP in A and C. Asterisks indicate a p-value of less than 0.005. **F.** Western analysis of Sp1 and Ets-1 expression in H1975 and KNS-62 cell lines stably expressing shRNA against the endogenous p53 mutant (or GFP) in the cells.

In order to decipher the mechanism used by GOF p53 in inducing upregulation of EGFR expression, we investigated the transcriptional machinery at the EGFR promoter which might be positively influenced by GOF p53. One such mechanism could be to promote chromatin opening through histone acetylation. To determine which TFs may be involved in influencing binding of AcH3 on the EGFR promoter, we transfected GOF p53-R273H expressing cells (and control) with individual siRNAs targeting different TFs (as well as a nonspecific control), and performed AcH3 ChIP to test if AcH3 binding on the EGFR promoter was changed along with decrease in the TF levels. If a particular TF satisfied this criterion, it would indicate the involvement of that particular TF in the acetylation of H3 histone binding on the EGFR promoter. Figure 6A depicts RNAi experimental data showing that siRNAs against CBP, Ets-1 and Sp1 had significant effects on binding of AcH3 on the EGFR promoter whereas RNAi against p63 had limited effects. Reduction of TF levels after treatment with the respective siRNA was confirmed by immunoblotting (Figure 6B). Thus, our results suggest involvement of Ets1, Sp1 and CBP in activation of the EGFR promoter by GOF p53.

### Sp1, CBP and Ets1 affect GOF p53 binding on the EGFR promoter

Since the data presented in Figure 6A indicated the involvement of TFs Ets1, Sp1 and the histone acetyl transferase (HAT) CBP, we wanted to test whether these factors are also required for interaction of GOF p53 on the promoter. To test this, once again we performed TF-directed RNAi experiments and carried out ChIP for GOF p53 to determine if lowering the levels of any of these TFs impacts GOF p53 binding. Figure 6C shows that although nonspecific siRNA did not affect the level of TFs or the binding of GOF p53 on the EGFR promoter, siRNA directed against Sp1, Ets1 and CBP significantly inhibited the interaction of p53-R273H with the EGFR promoter. Figure 6D shows that the individual transcription factor expression levels were indeed reduced without changing the level of GOF p53. This suggests that these TFs are involved in nucleating GOF p53 on the promoter. Transcription factor silencing also affects GOF p53-mediated EGFR protein expression (Figure 6D) and transcription (Figure 6E). To investigate whether reduction of mutant p53 would have an effect on the level of Sp1 or Ets-1 in lung cancer cells we performed Western analysis on H1975 and KNS-62 cells stably expressing shRNA against the endogenous mutant p53 (or GFP as control) and saw that when mutant p53 is knocked-down the level of Sp1, and to some degree Ets-1, is reduced as well (Figure 6F). This reduction of Sp1 is concordant with upregulation of Sp1 seen in H1299 cells expressing 273H *vs* vector in the immunoblot presented in Figure 6B.

### Facilitation of TF interactions on the EGFR promoter

**Figure 7:**
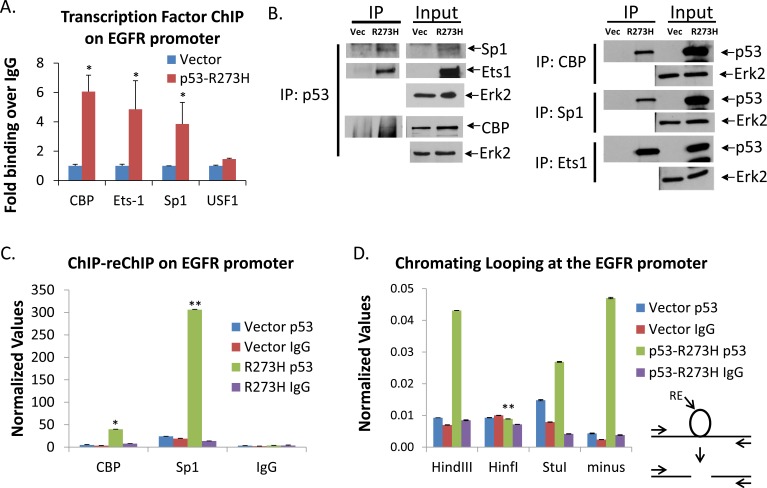
GOF p53 facilitates interaction of TFs on the EGFR promoter **A.** ChIP assays were carried out for individual TFs using specific antibodies in H1299 cells stably expressing p53-R273H (and vector control). QPCR was performed using gene specific primers. Data represent QPCR values normalized to a region on the GAPDH promoter which is not affected by GOF p53. Normalized values for each transcription factor were divided by the normalized IgG value to calculate fold binding over IgG. The vector was then set to 1 in each set to be able to compare binding between the different transcription factors. Experiments were performed in triplicate. Error bars showing standard deviations are indicated. Asterisks indicate a p-value of less than 0.05. ChIP of individual TFs on the EGFR promoter in the presence and absence of GOF p53 shows enhanced interaction of different TFs on the promoter. **B.** *In vivo* interactions between different TFs were carried out in H1299 cells stably expressing p53-R273H (or vector alone) without transfection of TFs by immunoprecipitation (IP) analysis. One set of immunoprecipitations was performed using an antibody against p53 and western blots were probed for Sp1, Ets1, and CBP; another set of immunoprecipitations was performed using antibodies against Sp1, Ets1, and CBP and then the western blots were probed for p53. Immunoprecipitation of p53-R273H from GOF p53 expressing cells shows binding of GOF p53 with CBP, Ets1, and Sp1. Erk2 is shown as a loading control for the IP inputs. **C.** ChIP-re-ChIP assay showing an increased interaction between GOF p53 and CBP as well as GOF p53 and Sp1. ChIP-re-ChIP procedures were carried out as described in Materials and Methods. Antibodies used for the first immunoprecipitation are indicated in the body of the figure, and antibodies used for the second immunoprecipitation are shown on the X-axis. Asterisks indicate a p-value of less than 0.05 for CBP and less than 0.005 for Sp1. **D.** Chromatin opening was tested by digestion of ChIP samples with restriction enzymes that are either present or absent in the EGFR promoter (and is described in Materials and Methods). ChIP was performed on H1299 cells stably expressing p53 mutant R273H (or vector control) using antibodies against p53 or IgG control and are indicated in the legend of the graph. The enzyme used for digestion is indicated on the X-axis. Primers used for PCR were located upstream and downstream of where restriction sites were located. Successful PCR indicated uncut chromatin while less or no PCR product indicated chromatin that was open for digestion. Digestion with HinfI and to some extent StuI prevented a PCR product from being formed. HindIII, which does not have a site within the promoter, was used as a digestion negative control. A schematic of the loop-digestion principle is shown. Experiments have been performed multiple times and similar results have been obtained. Asterisks indicate a p-value of less than 0.05.

We wanted to determine if GOF p53 facilitates interaction of one or more TFs on the EGFR promoter, and if there is any difference in interaction of TFs with the EGFR promoter in the presence of GOF p53. Therefore, we carried out ChIP assays as described [23] using antibodies against TFs with H1299 cell lines expressing p53-R273H and vector control. Figure 7A shows that GOF p53 induces an increased interaction of CBP, Ets1, and Sp1 on the EGFR promoter, suggesting cooperative interactions between these TFs and GOF p53.

*In vivo* GOF p53-TF interactions were studied by immunoprecipitation analysis using procedures described previously [34, 37]. Data shown in Figure 7B support the physical interaction of GOF p53 with Sp1, Ets1, and CBP. GOF p53-transcription factor cooperation is particularly high in cases of Ets1, Sp1 and CBP suggesting that GOF p53 induces nucleation of CBP on the EGFR promoter through Sp1 and Ets1. It is also possible that GOF p53 may stabilize or activate Ets1 and Sp1 and as a result up-regulate EGFR gene expression (Figure 7B).

We next used ChIP-re-ChIP experiments to determine which GOF p53-TF interactions are occurring on the chromatin itself. Figure 7C shows ChIP-re-ChIP data investigating the interaction of CBP and Sp1 and GOF p53 on the promoter, and demonstrates Sp1 as a strong candidate in multiple assays, while CBP also showed significant interactions on the promoter under the conditions of the assay. These data demonstrate that GOF p53 may nucleate on the EGFR promoter through Sp1 and, to some extent, CBP. Our data (Figure 7B and 7C) show GOF p53 binds with Sp1, Ets1, and CBP, supporting the above interactions.

### GOF p53 induces chromatin opening

To determine whether the chromatin was open at the EGFR promoter in the presence of GOF p53, we performed a chromatin loop assay. If the presence of GOF p53, and possibly other transcription factors, keeps the chromatin open for transcription, the DNA loop would be accessible for restriction enzyme digestion. We identified several restriction enzyme sites within a region of the EGFR promoter. The loop assay was performed as described in the Materials and Methods and PCR primers were used that were outside of the restriction sites. HinfI, StuI, and HindIII (negative restriction control) were used to digest DNA. Figure 7D shows that HinfI and to some extent StuI digestion cut the DNA loop which prevented a PCR product from being amplified. This loop assay data shows that the chromatin is open in the presence of GOF p53.

**Figure 8:**
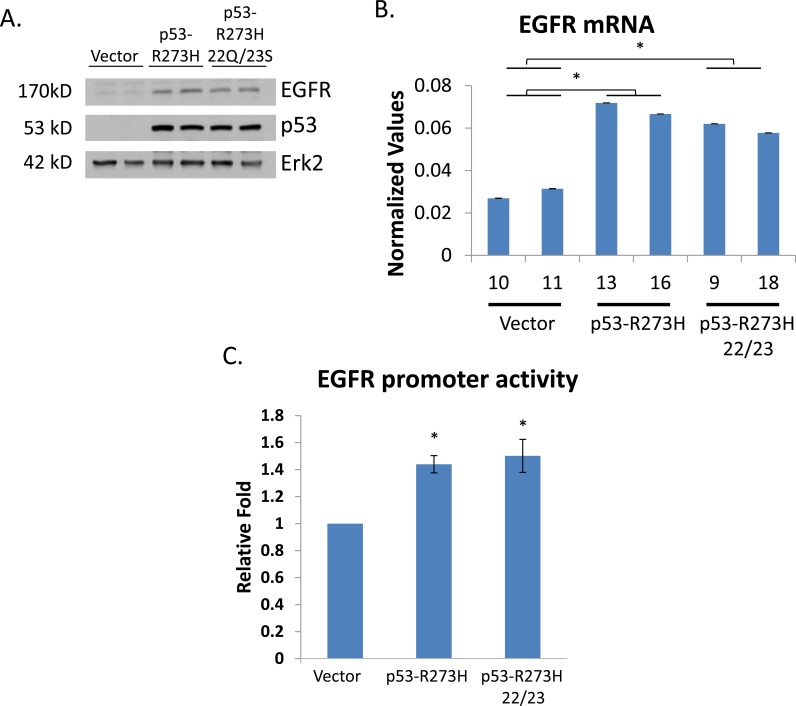
EGFR transactivation by GOF p53 can withstand mutations in the transactivation domain of GOF p53 H1299 cells were stably transfected with expression plasmids containing either the p53 mutant R273H, the p53 TAD mutant R273H (L22Q/W23S), or vector alone and cell clones were isloted. **A.** Western blots show expression levels of EGFR and mutant p53. **B.** RT-QPCR analysis showing levels of EGFR in different cell lines. QPCR was performed using gene specific primers. Normalized values represent QPCR values relative to GAPDH (not affected by GOF p53). Experiments were performed in triplicate. Error bars showing standard deviations are indicated. Asterisks indicate a p-value of less than 0.005. **C.** Luciferase assay showing transactivation of the EGFR promoter by both p53-R273H as well as p53-R273H L22Q/W23S. Asterisks indicate a p-value of less than 0.05.

EGFR transactivation by GOF p53 can withstand mutations in the p53 transactivation domain. To determine if the transactivation domain (TAD) of GOF p53 is needed for up-regulation of EGFR expression, we stably transfected H1299 cells with p53-R273H or p53-R273H (L22Q/W23S) (or vector alone) and isolated independent cell clones. Figure 8 shows Western blot analysis of EGFR and p53 levels of the clones used in our assays. Next, we isolated RNA from each cell clone, and performed quantitative RT-QPCR to determine the level of EGFR mRNA in the different cell lines. The data presented in Figure 8B show that the TAD mutations did not affect EGFR induction by GOF p53-R273H. To ensure that our TAD mutants do in fact transactivate the EGFR promoter we performed a transient transactivation assay and found (Figure 8C) that the presence of TAD mutations do not inhibit EGFR promoter activation.

### TAD mutations differentially affect GOF p53 interactions and binding of acetylated histones on the EGFR promoter

**Figure 9:**
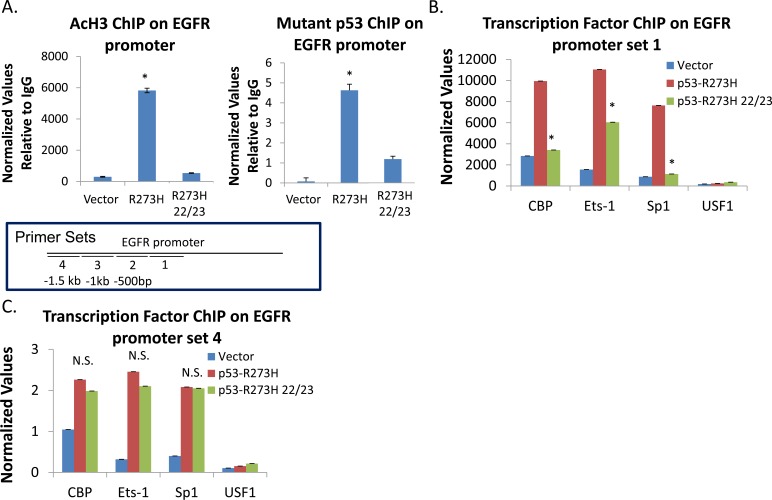

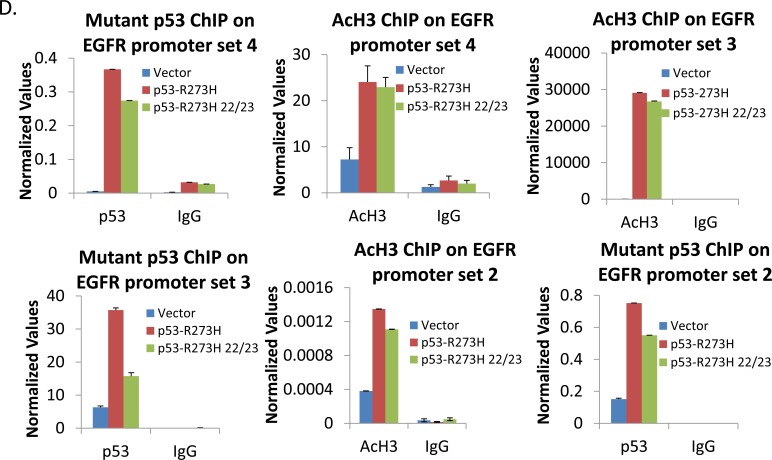
TAD mutations differentially affect GOF p53 interactions and binding of acetylated histones with the EGFR promoter **A.** ChIP assays showing mutations in TAD alter a majority of interactions of GOF p53 and enhanced binding of acetylated histone H3 to the EGFR promoter. Experiments were carried out as described in Materials and Methods. Data represent QPCR values normalized to a region on the GAPDH promoter which is not affected by GOF p53. Normalized values were divided by the normalized IgG. Error bars showing standard deviations are indicated. Asterisks indicate a p-value of less than 0.05. **B.** ChIP assays showing mutations in TAD alter binding of different transcription factors to the EGFR promoter. Experiments were carried out as described in Materials and Methods. Data represent QPCR values normalized to a region on the GAPDH promoter which is not affected by GOF p53. Error bars showing standard deviations are indicated. Asterisks indicate a p-value of less than 0.05. **C.** ChIP samples in Figure 9B were assayed using a different set of primers on the EGFR promoter about 1.5kb upstream of set 1 to show GOF p53 and its TAD mutant have a similar binding pattern at a distant location (as shown in the diagram). Data represent QPCR values normalized to a region on the GAPDH promoter which is not affected by GOF p53. Error bars showing standard deviations are indicated. N.S. indicates no significant difference. **D.** ChIP samples in Figure 9A were assayed using three different sets of primers on the EGFR promoter about 500bp-1.5kb upstream of set 1 to show GOF p53 and its TAD mutant have a similar binding pattern at a distant locations. Positions of the primer sets are shown in the figure. Data represent QPCR values normalized to a region on the GAPDH promoter which is not affected by GOF p53. Error bars showing standard deviations are indicated. N.S. indicates no significant difference.

Since TAD is an important component of the transactivation machinery and is where TFs have a tendency to contact p53 [38], we wanted to test if TAD mutations affect transactivation by GOF p53 *via* effects on GOF p53 binding on the EGFR promoter and/or effects on histone acetylation. Therefore, we used QPCR to quantitatively determine the effects of TAD mutations on GOF p53-mediated activation of EGFR transcription (assayed by RT-QPCR) as well as nucleation of p53-R273H and AcH3 on its promoters (assayed by ChIP). TAD mutations did indeed show a significant reduction of GOF p53 and TF interactions on the region examined (shown in Figure 5B). This is accompanied by a reduction of histone H3 acetylation (Figure 9A). Next we looked at the ability of our TAD mutant cell line to recruit transcription factors to the EGFR promoter. Figure 9B shows a reduction of transcription factor binding that was similar to the reduction of GOF p53 and histone H3 acetylation binding in Figure 9A at the region shown in Figure 5B. As our experiments (Figure 8) indicated that the TAD mutant is able to stimulate EGFR transcription as efficiently as the R273H mutant, this suggested to us that GOF p53 with TAD mutations might be efficiently interacting at one or more different sites other than that shown in Figure 5B. We performed ChIP using PCR primers corresponding to sequences spanning different regions on the EGFR promoter as shown on the schematic. Figure 9D shows data in support of our hypothesis that GOF p53-R273H and its TAD mutant both interact significantly with multiple locations on the EGFR promoter (identified by ChIP Seq), although the TAD mutant failed to interact in the primary site identified by ChIP Seq. Taken together, the data indicate that these interactions result in activation of histone H3 acetylation where TFs are also successfully recruited (Figure 9C). It is possible that TAD interacts with different sequences of the EGFR promoter region using motifs defined by amino acids other than those mutated in the present construct (amino acids 22 and 23).

## DISCUSSION

Our data show that lung cancer cells with endogenous GOF p53 are addicted to GOF p53 (Figure 4A), and one pathway used in the addiction is the EGFR pathway as the defect can be rectified by overexpressing EGFR in H1975 cells (Figure 4D, 4G). Thus, our data establish a connection between addiction of lung cancer cells to GOF p53 and the cellular EGFR pathway.

Here we examined the mechanism of transactivation of the EGFR gene by GOF p53, found unique features, and identified a sequence element that responds to GOF p53 (Figure 5G, 5H). We also investigated the mechanism of up-regulation of EGFR expression by GOF p53 and demonstrated that EGFR is elevated at the mRNA level by GOF p53 in H1299, KNS-62 and H1975 cells. Through RNAi experiments in H1975 cells we show that reduction of GOF p53 or EGFR levels lowers the proliferation rate of these cells, indicating that both genes are in a pathway that controls cell proliferation. Since GOF p53 up-regulates EGFR, this also suggests that this particular function of GOF p53 is through EGFR up-regulation, at least in part. This concept has been strengthened further by restoration of GOF activity lost by reduction of p53 levels on over-expression of EGFR (Figure 4). However, multiple GOF p53 targets have been identified by us and others [23, 39, 40] that may be responsible for induction of proliferation.

We also show that GOF p53 expression leads to enhanced binding of GOF p53 on the EGFR promoter, and importantly it induces enhanced interaction of TFs on the EGFR promoter including CBP. ChIP for AcH3 indicates enhanced acetylation of histone H3 in the presence of GOF p53, indicative of induced opening of the chromatin near the GOF p53 binding site. Thus, the mechanism by which GOF p53 activates EGFR transcription may depend upon nucleation of GOF p53 that then induces acetylation of histone H3, opening chromatin and activating transcription.

We examined the contribution of TAD-I in transactivation of EGFR and nucleation of GOF p53 and AcH3 (as assayed by ChIP) on the EGFR promoter. Interestingly, mutations in TAD-I affected nucleation of GOF p53 and AcH3 at the major binding site (Figure 9) but not transactivation of EGFR (Figure 8). Since our data show no absolute requirements of integrity of amino acids at positions 22 and 23 for EGFR promoter activation, it perhaps points to the possible interactions of TFs included in extended regions of TAD. However, by deletion analysis we showed earlier that transactivation of the EGFR promoter by GOF p53 requires the presence of TAD [18], suggesting an important contribution of TAD in transactivation. The scanning ChIP QPCR data shown in Figure 9 suggest that the promoter sequences defined by primer set 1 requires the integrity of amino acids 22 and 23 of TAD (TAD-I, to be precise), perhaps through a direct interaction of TFs whereas TAD can contact other regions *via* other TAD sequences. It is possible in that case sequences in the TAD-II region also come into play [38]. Thus, the EGFR promoter is a GOF p53 TAD-I mutation resistant promoter and multiple contacts of the promoter occur with one or more TF with GOF p53 in a TAD-I independent manner. In the future it would be interesting to mechanistically test a number of TAD-I sensitive and resistant promoters as well to determine if multiple contacts are seen in all of them.

It is important to note that we find GOF p53 induces enhanced interactions of TFs on the EGFR promoter. It is possible that this results in enhanced binding of CBP/p300 to the EGFR promoter and consequently higher levels of acetylation of histone H3. This is expected to impact the chromatin structure in a positive manner, paving the way for a higher rate of transcription. In the future, it needs to be ascertained if the information exchange between the proteins happens while they remain on the promoter or when they are unbound from the DNA. Involvement of the Sp1 and Ets group of transcription factors and GOF p53 in GOF p53-mediated transactivation as a component of its GOF activity has been suggested by us and others in the past [41, 42]. As we saw in Figure 6B and 6F, Sp1 and Ets-1 are upregulated by GOF p53. When we knocked-down expression of endogenous mutant p53 in H1975 and KNS-62, expression of Sp1 and Ets-1 was reduced. This reduction of protein levels of TFs may explain why transcription of certain genes is inhibited in shp53 cells that are upregulated by GOF p53.

**Figure 10:**
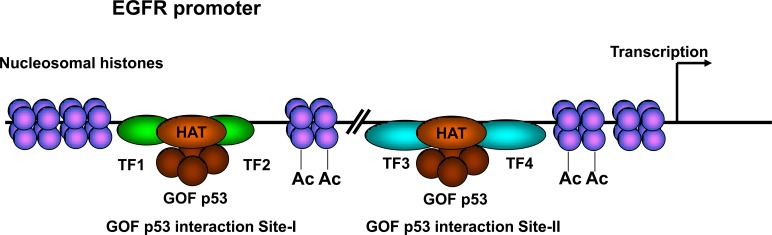
Proposed model for GOF p53 nucleating on the EGFR promoter Arrow towards the right hand side depicts transcription direction. The model proposes a single tetramer of GOF p53 to interact with multiple TFs resulting in nucleation of HAT. Two GOF p53 interaction sites are shown, one is sensitive to TAD-I mutations, the other is not. Empirically, Site I has been shown with one set of transcription factors and Site II with with a different set. TF1/2 and TF3/4 could be similar or different, but presumably TF1/2 is different from TF3/4. For simplicity, TAF and other factors are not shown. TF = transcription factor.

We suggest a model (Figure 10) in which GOF p53 interacts on the EGFR promoter *via* multiple TFs: Ets1, Sp1 and perhaps others (Figures 6 and 7); possibly, GOF p53 docks with Sp1 and CBP (Figure 7C). p300/CBP may become involved in the process either because of direct interaction of p53 and CBP/p300 or through Ets1-CBP/p300 and/or Sp1-CBP/p300 interactions [43-46]. Sp1 and Ets1 interactions with CBP/p300 have been suggested to facilitate acetylation of histones [47, 48]. This increased histone acetylation then translates into chromatin opening and increased transcription. We have tested the presence of chromatin opening through a new chromatin loop assay (Figure 7D) and were able to show reduction of PCR product formation after digestion of DNA indicating that the chromatin was in an open conformation and accessible for restriction enzyme digestion.

Our work shows a possible pathway used by GOF p53 to establish addiction of lung cancer cells. It might be possible to intervene in the EGFR-GOF p53 pathway for lung cancers showing over-expression of EGFR, particularly if they harbor GOF p53. Thus, this could open up a new therapeutic angle.

## MATERIALS AND METHODS

### Cells

H1299, H1975 and KNS-62 cell lines were purchased from commercial sources and were maintained in media as suggested by the suppliers. Methods for lipofection, nucleofection, and generation of stable transfectants were as described [21-23]. Clones were isolated using puromycin selection at 1μg/ml or G418 at 400 μg/ml.

### Generation of H1299 cells expressing GOF p53 mutants

To determine the influence of the transactivation domains on GOF p53-mediated transactivation, we constructed 3 amino acid substitution mutants: p53-R273H (L22Q/W23S) using the Quikchange mutagenesis kit (Agilent; Santa Clara, CA), sequence verified the plasmid clone and expressed these in H1299 cells. Multiple clones were isolated with p53 expression approximately equivalent to that of p53-R273H alone. We used these clones in comparison with vector transfected cell clones for our assays. We have also used H1299 cells expressing different p53 mutants as described earlier [9].

### EGFR promoter transient assays

The EGFR promoter-luciferase construct was obtained from Active Motif (Carlsbad, CA). The EGFR expression plasmid was created by cloning the EGFR cDNA sequence into the pWZL Hygro plasmid purchased from Addgene (Cambridge, MA). Similar constructs have also been made in pMSCV-IRES-mCherry FP vector (Addgene, Dario Vignali, unpublished) replacing SpeI EcoRI fragment with XbaI- KpnI fragment containing the EGFR-luc in pGL4 luc (Promega). These constructs respond to GOF p53 showing enhanced red fluorescence and luciferase activity. Mouse lung cells with p53 −/− and R172H/R172H were plated on coverslips, transfected with the mCherry-EGFR.luc expression plasmid, and mounted on slides after fixation [24, 25]. Cells were viewed under a Zeiss LSM 700 confocal microscope using the 20X objective. Luciferase analysis was carried out using the dual luciferase assay system (E1500) and instructions from Promega. Transient transfection was performed with 100ng of promoter and 50ng of expression plasmid using Lipofectamine 3000 (Invitrogen) following the manufacturer's instructions. Both transfection and luciferase assays were performed as described previously in triplicate [26].

### siRNA transfection

siRNAs were nucleofected into H1299 cells expressing p53-R273H or vector control following the manufacturer's instructions (Lonza; Walkersville, MD). Sequences used to target individual transcription factors were as follows: siCBP: 5′-UUGAGGAAUCAACAGCCGCtt-3′ [27], siEGFR: 5′-GCAAAGUGUGUAACGGAAUAGGUAUtt-3′ [28], siEts1: 5′-ACUUGCUACCAUCCCGUACtt-3′ [29], sip63: 5′-AAAGCAGCAAGUUUCGGACAGtt-3′ [30], siSp1: 5′-GGUAGCUCUAAGUUUUGAUtt-3′ [31], and siScrambled (control): 5′-CAUGUCAUGUGUCACAUUCtt-3′ [32].

### Growth assays

Growth assays were carried out as described by us earlier with slight modifications [9]. Cells were plated at a density of 50,000 cells/6cm dish in triplicate for five time points, harvested after incubation with trypsin and counted using a Coulter Counter (Beckman). For gene knock-down studies, siRNA transfection was carried out for two consecutive days before starting the growth assay. All experiments were performed in triplicate.

### Xenograft assay

Nu/J (Nude; Jackson Labs, Bar Harbor, ME) or NOD.CB17-*Prkdc^scid^*/NcrCrl (Scid; Charles River Labs, Raleigh, NC) mice were used for the tumorigenicity studies. Mice were injected with 1×10^7^ cells subcutaneously on the flanks and tumors allowed to grow to a maximum size of 1cm, measuring periodically as described before [22]. At least two different clones of cells were used to rule out clonal variations. For the xenograft assays where transfections were done prior to injection, we counted the number of cells after transfection at the day of injection (48-72h post transfection).

### Western blotting

Immunoblotting was carried out as described earlier [9]. Briefly, for a typical Western blot, extracts were prepared in Promega Lysis Buffer (Promega). For immunoblots to detect phosphorylated proteins, extracts were prepared in RIPA buffer (see below) with the addition of phosphatase inhibitors (Phosphatase Inhibitor Cocktail 3; P0044, Sigma Aldrich and Halt Phosphatase Inhibitor Cocktail; 1862495, Thermo Fisher Scientific). p53 was detected using the p53 antibody PAb 1801 (93), EGFR and Erk2 antibodies were from Santa Cruz Biotechnology (Dallas, TX) (sc-03 and sc-154 respectively), phospho-EGFR was from Cell Signaling (Danvers, MA) (2234); transcription factors (TFs) were detected using respective antibodies from Santa Cruz Biotechnology: CBP (sc-369), Ets-1 (sc-350), p63 (sc-8431), and Sp1 (sc-59). Western blots were developed by the ECL method (GE Healthcare; Piscataway, NJ).

### Tumor RNA analysis and p53 sequencing

Tumor RNAs were provided by the Tissue and Data Acquisition and Analysis Core repository under an Institutional Review Board approved protocol (HM12985); cDNAs were prepared using the Superscript III cDNA synthesis kit (Invitrogen) and QPCR performed using primers specific for EGFR (F: 5′-AAGTGTAAGAAGTGCGAAGG-3′ and R: 5′-GGAGGAGTATGTGTGAAGGA-3′). The degree of expression was quantified using a relative standard curve and normalized to GAPDH (F: 5′-GTCAACGGATTTGGTCGTATT-3′ and R: 5′-GATCTCGCTCCTGGAAGATGG-3′) corresponding to the cDNA batch. The p53 gene was sequenced as described previously [23]. Whenever a mutation was found, a new PCR reaction was performed and the amplified fragment re-sequenced to verify the previous result.

### Chromatin immunoprecipitation

Chromatin immunoprecipitation (ChIP) assays were performed as described earlier [9]. Antibodies used for ChIP were: p53 (DO1: sc-126 and FL-393: sc-6243, Santa Cruz), acetylated histone H3 that recognizes acetylated lysine at positions 9 and 14 (17-615, Millipore; Billerica, MA), TFs (CBP (sc-369), Ets-1 (sc-350), p63 (sc-8431), Sp1 (sc-59), and USF1 (sc-229), Santa Cruz Biotechnology) and IgG (normal mouse: sc-2025 and normal rabbit: sc-2027, Santa Cruz). Quantitative PCR (QPCR) was used to quantify precipitated DNA using promoter specific primers. The following primers were used: GAPDH ChIP (F: 5′-GTATTCCCCCAGGTTTACAT-3′ and R: 5′-TTCTGTCTTCCACTCACTCCT-3′), EGFR ChIP set 1 (F: 5′-CCCGCGCGAGCTAGACGTCC-3′ and R: 5′-GCTCGCTCCGGCTCTCCC-3′), EGFR ChIP set 2 (F: 5′-ACTATGAAGGCTGTTGTCTC-3′ and R: 5′-ACAACAGTGGAACATAAAAT-3′), EGFR ChIP set 3 (F: 5′-TCTGTGTTTCTACGGACTGC-3′ and R: 5′-ATGTTTGTGCCTGGGTCT-3′), and EGFR ChIP set 4 (F: 5′-AAAGATGTAAGGTTGCTCCC-3′ and R: 5′-TTGGCCAAAAGAAACTGAG-3′). ChIP-re-ChIP was performed following the method described [33] by incubating equal amounts of extracts with p53 antibodies or control IgG overnight and then incubating with BSA and sonicated salmon sperm saturated protein A agarose beads for one hour at 4°C. The DNA-protein-antibody complexes were then washed once with RIPA (150mM NaCl, 50mM Tris pH8, 0.1% SDS, 0.5% sodium deoxycholate, 1% NP-40), once with High Salt Buffer (500mM NaCl, 50mM Tris pH 8, 0.1% SDS, 1% NP-40), once with LiCl Buffer (250mM LiCl, 50mM Tris pH 8, 0.5% sodium deoxycholate, 1% NP-40), and once with 1X TE. DNA-protein complexes were eluted from the protein A agarose beads by incubation at 37°C for 30min in 10mM DTT in 1X TE. Eluants were then diluted 1:20 and incubated with the indicated second antibody overnight, and BSA and sonicated salmon sperm saturated protein A agarose beads were added for one hour at 4°C the following day. The DNA-protein-antibody complexes were then washed once with RIPA, once with High Salt Buffer, once with LiCl Buffer, and once with 1X TE. DNA-protein complexes were eluted at 65°C overnight in fresh elution buffer (20% SDS, 10mM DTT, 100mM NaHCO_3_), RNase and proteinase K digested, phenol/chloroform extracted, and QPCR was performed with specific primers.

### Chromatin loop assay

Samples were prepared for ChIP, immunoprecipitated, and washed as described above. Equal amounts of extracts were incubated with p53 antibody or control IgG overnight and then incubated with BSA and sonicated salmon sperm saturated protein A agarose beads for one hour at 4°C. The DNA-protein-antibody complexes were then washed once with RIPA, once with High Salt Buffer, twice with LiCl Buffer, and twice with 1X TE. After washing, DNA-protein complexes bound to the protein A agarose beads were incubated with specific restriction enzymes at 37°C for one hour and then the DNA-protein complexes were eluted from the beads at 65°C overnight in fresh elution buffer (20% SDS, 10mM DTT, 100mM NaHCO_3_), RNase and proteinase K digested, phenol/chloroform extracted, and QPCR was performed with specific primers on either side of the loop.

### Immunoprecipitation assays

Co-immunoprecipitation (Co-IP) of proteins as an indication of protein-protein interactions was carried out as described earlier [34, 35]. Briefly, cells were washed with 1X PBS and harvested in NP-40 Buffer (50mM Tris pH 7.5, 150mM NaCl, 2mM EDTA, 0.5% NP-40 supplemented with PMSF and protease inhibitors). Cells were lysed for 30min on ice and passaged through a 27G needle three times. Lysates were centrifuged and protein concentrations were determined using the BCA Protein Assay Kit (Thermo Scientific; Waltham, MA). Equal protein amounts were used for IP. Protein extracts were precleared with protein A agarose rocking at 4°C for one hour. The extract/bead mix was centrifuged and the supernatant was transferred to new tubes. Extracts were then incubated with an antibody against p53 (PAb 421), CBP (sc-369, Santa Cruz), Sp1 (sc-59, Santa Cruz), or Ets1 (sc-350, Santa Cruz) and protein A agarose beads while rocking at 4°C overnight. The following morning the extract/bead/antibody mix was centrifuged and the beads were washed three times with NP-40 Buffer. The buffer was removed and equal volume 2X Laemmli loading buffer was added and boiled for ten minutes. Extracts were then resolved by sodium dodecyl sulfate polyacrylamide gel electrophoresis (SDS-PAGE). Additionally, a small aliquot of the IP supernatant was set aside and co-electrophoresed as a loading control.

### Cell migration assays

Cell migration was determined by wound closure assays described previously [10]. Briefly, cells were trypsinized, counted, plated in both chambers of tissue culture inserts (Ibidi GmbH, Martinsried, Germany), and then grown to confluence. The insert was removed, and the distance across the cell-free zone measured (Axiovision software; Carl Zeiss Microimaging, Thornwood, NY). Cultures were returned to the incubator, allowed to migrate for 8h, and the width of the cell-free zone re-measured. Migration rate was determined by subtraction of the final measurement of distance from the initial measurement, divided by time.

### Statistical analysis

All statistical analyses were calculated using the student's *t*-test. Data were considered significant if the p-value was below 0.05.

## References

[1] Chang YL, Wu CT, Shih JY, Lee YC (2011). Comparison of p53 and epidermal growth factor receptor gene status between primary tumors and lymph node metastases in non-small cell lung cancers. Ann Surg Oncol.

[2] Harris CC (1996). p53 tumor suppressor gene: from the basic research laboratory to the clinic—an abridged historical perspective. Carcinogenesis.

[3] El-Deiry WS (2003). The role of p53 in chemosensitivity and radiosensitivity. Oncogene.

[4] Bristow RG, Peacock J, Jang A, Kim J, Hill RP, Benchimol S (2003). Resistance to DNA-damaging agents is discordant from experimental metastatic capacity in MEF ras-transformants-expressing gain of function MTp53. Oncogene.

[5] Lin J, Teresky AK, Levine AJ (1995). Two critical hydrophobic amino acids in the N-terminal domain of the p53 protein are required for the gain of function phenotypes of human p53 mutants. Oncogene.

[6] Lanyi A, Deb D, Seymour RC, Ludes-Meyers JH, Subler MA, Deb S (1998). ‘Gain of function’ phenotype of tumor-derived mutant p53 requires the oligomerization/nonsequence-specific nucleic acid-binding domain. Oncogene.

[7] Scian MJ, Stagliano KE, Anderson MA, Hassan S, Bowman M, Miles MF, Deb SP, Deb S (2005). Tumor-derived p53 mutants induce NF-kappaB2 gene expression. Molecular and cellular biology.

[8] Blandino G, Levine AJ, Oren M (1999). Mutant p53 gain of function: differential effects of different p53 mutants on resistance of cultured cells to chemotherapy. Oncogene.

[9] Scian MJ, Stagliano KE, Ellis MA, Hassan S, Bowman M, Miles MF, Deb SP, Deb S (2004). Modulation of gene expression by tumor-derived p53 mutants. Cancer Res.

[10] Yeudall WA, Vaughan CA, Miyazaki H, Ramamoorthy M, Choi MY, Chapman CG, Wang H, Black E, Bulysheva AA, Deb SP, Windle B, Deb S (2012). Gain-of-function mutant p53 upregulates CXC chemokines and enhances cell migration. Carcinogenesis.

[11] Zalcenstein A, Stambolsky P, Weisz L, Muller M, Wallach D, Goncharov TM, Krammer PH, Rotter V, Oren M (2003). Mutant p53 gain of function: repression of CD95(Fas/APO-1) gene expression by tumor-associated p53 mutants. Oncogene.

[12] Weisz L, Damalas A, Liontos M, Karakaidos P, Fontemaggi G, Maor-Aloni R, Kalis M, Levrero M, Strano S, Gorgoulis VG, Rotter V, Blandino G, Oren M (2007). Mutant p53 enhances nuclear factor kappaB activation by tumor necrosis factor alpha in cancer cells. Cancer Res.

[13] Moll UM, Zaika A (2001). Nuclear and mitochondrial apoptotic pathways of p53. FEBS Lett.

[14] Zhou G, Wang J, Zhao M, Xie TX, Tanaka N, Sano D, Patel AA, Ward AM, Sandulache VC, Jasser SA, Skinner HD, Fitzgerald AL, Osman AA, Wei Y, Xia X, Songyang Z (2014). Gain-of-function mutant p53 promotes cell growth and cancer cell metabolism *via* inhibition of AMPK activation. Mol Cell.

[15] Sigal A, Rotter V (2000). Oncogenic mutations of the p53 tumor suppressor: the demons of the guardian of the genome. Cancer Res.

[16] Zalcenstein A, Weisz L, Stambolsky P, Bar J, Rotter V, Oren M (2006). Repression of the MSP/MST-1 gene contributes to the antiapoptotic gain of function of mutant p53. Oncogene.

[17] Deb SP, Munoz RM, Brown DR, Subler MA, Deb S (1994). Wild-type human p53 activates the human epidermal growth factor receptor promoter. Oncogene.

[18] Ludes-Meyers JH, Subler MA, Shivakumar CV, Munoz RM, Jiang P, Bigger JE, Brown DR, Deb SP, Deb S (1996). Transcriptional activation of the human epidermal growth factor receptor promoter by human p53. Molecular and cellular biology.

[19] Oda K, Matsuoka Y, Funahashi A, Kitano H (2005). A comprehensive pathway map of epidermal growth factor receptor signaling. Mol Syst Biol.

[20] Bethune G, Bethune D, Ridgway N, Xu Z (2010). Epidermal growth factor receptor (EGFR) in lung cancer: an overview and update. J Thorac Dis.

[21] Frum R, Ramamoorthy M, Mohanraj L, Deb S, Deb SP (2009). MDM2 controls the timely expression of cyclin A to regulate the cell cycle. Mol Cancer Res.

[22] Vaughan CA, Singh S, Windle B, Sankala HM, Graves PR, Andrew Yeudall W, Deb SP, Deb S (2012). p53 mutants induce transcription of NF-kappaB2 in H1299 cells through CBP and STAT binding on the NF-kappaB2 promoter and gain of function activity. Arch Biochem Biophys.

[23] Vaughan CA, Singh S, Windle B, Yeudall WA, Frum R, Grossman SR, Deb SP, Deb S (2012). Gain-of-Function Activity of Mutant p53 in Lung Cancer through Up-Regulation of Receptor Protein Tyrosine Kinase Axl. Genes Cancer.

[24] Lang GA, Iwakuma T, Suh YA, Liu G, Rao VA, Parant JM, Valentin-Vega YA, Terzian T, Caldwell LC, Strong LC, El-Naggar AK, Lozano G (2004). Gain of function of a p53 hot spot mutation in a mouse model of Li-Fraumeni syndrome. Cell.

[25] Donehower LA, Harvey M, Slagle BL, McArthur MJ, Montgomery CA, Butel JS, Bradley A (1992). Mice deficient for p53 are developmentally normal but susceptible to spontaneous tumours. Nature.

[26] Vaughan CA, Singh S, Windle B, Sankala HM, Graves PR, Andrew Yeudall W, Deb SP, Deb S p53 mutants induce transcription of NF-kappaB2 in H1299 cells through CBP and STAT binding on the NF-kappaB2 promoter and gain of function activity. Arch Biochem Biophys.

[27] Ma H, Nguyen C, Lee KS, Kahn M (2005). Differential roles for the coactivators CBP and p300 on TCF/beta-catenin-mediated survivin gene expression. Oncogene.

[28] Chen G, Kronenberger P, Teugels E, Umelo IA, De Greve J (2012). Targeting the epidermal growth factor receptor in non-small cell lung cancer cells: the effect of combining RNA interference with tyrosine kinase inhibitors or cetuximab. BMC Med.

[29] Ni W, Zhan Y, He H, Maynard E, Balschi JA, Oettgen P (2007). Ets-1 is a critical transcriptional regulator of reactive oxygen species and p47(phox) gene expression in response to angiotensin II. Circ Res.

[30] Barbieri CE, Tang LJ, Brown KA, Pietenpol JA (2006). Loss of p63 leads to increased cell migration and up-regulation of genes involved in invasion and metastasis. Cancer Res.

[31] Jungert K, Buck A, von Wichert G, Adler G, Konig A, Buchholz M, Gress TM, Ellenrieder V (2007). Sp1 is required for transforming growth factor-beta-induced mesenchymal transition and migration in pancreatic cancer cells. Cancer Res.

[32] Vaughan C, Mohanraj L, Singh S, Dumur CI, Ramamoorthy M, Garrett CT, Windle B, Yeudall WA, Deb S, Deb SP (2011). Human Oncoprotein MDM2 Up-regulates Expression of NF-kappaB2 Precursor p100 Conferring a Survival Advantage to Lung Cells. Genes Cancer.

[33] Furlan-Magaril M, Rincon-Arano H, Recillas-Targa F (2009). Sequential chromatin immunoprecipitation protocol: ChIP-reChIP. Methods Mol Biol.

[34] Singh S, Ramamoorthy M, Vaughan C, Yeudall WA, Deb S, Palit Deb S (2013). Human oncoprotein MDM2 activates the Akt signaling pathway through an interaction with the repressor element-1 silencing transcription factor conferring a survival advantage to cancer cells. Cell Death Differ.

[35] Brown DR, Deb S, Munoz RM, Subler MA, Deb SP (1993). The tumor suppressor p53 and the oncoprotein simian virus 40 T antigen bind to overlapping domains on the MDM2 protein. Molecular and cellular biology.

[36] Vaughan CA, Deb SP, Deb S, Windle B (2014). Preferred binding of gain-of-function mutant p53 to bidirectional promoters with coordinated binding of ETS1 and GABPA to multiple binding sites. Oncotarget.

[37] Leng P, Brown DR, Shivakumar CV, Deb S, Deb SP (1995). N-terminal 130 amino acids of MDM2 are sufficient to inhibit p53-mediated transcriptional activation. Oncogene.

[38] Freed-Pastor WA, Mizuno H, Zhao X, Langerod A, Moon SH, Rodriguez-Barrueco R, Barsotti A, Chicas A, Li W, Polotskaia A, Bissell MJ, Osborne TF, Tian B, Lowe SW, Silva JM, Borresen-Dale AL (2012). Mutant p53 disrupts mammary tissue architecture *via* the mevalonate pathway. Cell.

[39] Oren M, Rotter V (2010). Mutant p53 gain-of-function in cancer. Cold Spring Harb Perspect Biol.

[40] Freed-Pastor WA, Prives C (2012). Mutant p53: one name, many proteins. Genes & development.

[41] Do PM, Varanasi L, Fan S, Li C, Kubacka I, Newman V, Chauhan K, Daniels SR, Boccetta M, Garrett MR, Li R, Martinez LA (2012). Mutant p53 cooperates with ETS2 to promote etoposide resistance. Genes & development.

[42] Sampath J, Sun D, Kidd VJ, Grenet J, Gandhi A, Shapiro LH, Wang Q, Zambetti GP, Schuetz JD (2001). Mutant p53 cooperates with ETS and selectively up-regulates human MDR1 not MRP1. The Journal of biological chemistry.

[43] Foulds CE, Nelson ML, Blaszczak AG, Graves BJ (2004). Ras/mitogen-activated protein kinase signaling activates Ets-1 and Ets-2 by CBP/p300 recruitment. Molecular and cellular biology.

[44] Yang C, Shapiro LH, Rivera M, Kumar A, Brindle PK (1998). A role for CREB binding protein and p300 transcriptional coactivators in Ets-1 transactivation functions. Molecular and cellular biology.

[45] Nelson ML, Kang HS, Lee GM, Blaszczak AG, Lau DK, McIntosh LP, Graves BJ (2010). Ras signaling requires dynamic properties of Ets1 for phosphorylation-enhanced binding to coactivator CBP. Proceedings of the National Academy of Sciences of the United States of America.

[46] Huang W, Zhao S, Ammanamanchi S, Brattain M, Venkatasubbarao K, Freeman JW (2005). Trichostatin A induces transforming growth factor beta type II receptor promoter activity and acetylation of Sp1 by recruitment of PCAF/p300 to a Sp1. NF-Y complex. The Journal of biological chemistry.

[47] Das C, Lucia MS, Hansen KC, Tyler JK (2009). CBP/p300-mediated acetylation of histone H3 on lysine 56. Nature.

[48] Henry RA, Kuo YM, Andrews AJ (2013). Differences in specificity and selectivity between CBP and p300 acetylation of histone H3 and H3/H4. Biochemistry.

